# Characterization of the complete mitochondrial genome of the endangered species *Schizothorax integrilabiatus*

**DOI:** 10.1080/23802359.2018.1445486

**Published:** 2018-02-28

**Authors:** Chi Zhang, Bo Ma, Mey Gyurmey, Haiping Liu, Wanliang Wang, Baohai Li, Bianbian Zhang

**Affiliations:** a Institute of Fisheries Science, Tibet Academy of Agricultural and Animal Husbandry Sciences, Lhasa, PR China;; b Ministry of Agriculture of China, Heilongjiang Fisheries Research Institute, Chinese Academy of Fishery Sciences, Haerbin, PR China;; c Agriculture and Animal Husbandry Bureau, Tibetan Autonomous Region Motuo County, Motuo, PR China

**Keywords:** *Schizothorax integrilabiatus*, mitochondrial genome, phylogenetic

## Abstract

*Schizothorax integrilabiatus* is an endangered fish species found in the Buqun Lake of Qinghai-Tibet Plateau. In this study, we determined the complete mitochondrial genome sequence of the S*. integrilabiatus*. The circular mitochondrial genome was 16,621 bp in length, containing 13 protein-coding genes (PCGs), 22 transfer RNA (tRNA) genes, two ribosomal RNA (rRNA) genes and a control region (D-loop). The overall base composition is A 30.1%, C 26.9%, G 17.4%, and T 25.6%, with a high A + T content (55.7%). Further, phylogenetic analysis suggested that *S. integrilabiatus* is closely related to species of *S. plagiostomus*, and then clustered into a clade with other Schizothoracinae species. This work provides additional molecular information for studying *S. integrilabiatus* conservation genetics and evolutionary relationships.

Species richness is influenced by the environmental characteristics of habitat geomorphology, climate and species interactions (Kang et al. 2014). The Qinghai-Tibet Plateau Region is a special area that have a variety of ecosystems, a few species such as the *Schizothorax integrilabiatus* is distributed only in this area in the world (Wu and Wu 1992). Compared with other Schizothoracinae fishes, *S. integrilabiatus* distribution area is more narrow and population is smaller (Zhang et al. 1995). Based on the very small extent of occurrence and the potentially severe threat of fishing mortality, this species is defined as a critically endangered species by IUCN Red List of Threatened Species (IUCN 2010). Therefore, we sequence and annotate the mitochondrial genome of this fish, which has important research significance in species evolution and resource protection.

The specimens were obtained from the Buqun Lake (29°15′N;95°13′E) at an altitude of 1545 m in 2015. A 30–40 mg fn clip was collected and preserved in the Fisheries Research Institute, Tibet Academy of Agricultural and Animal Husbandry Sciences. The total genomic DNA was extracted from the pelvic fin preserved in 95% alcohol. The genomic DNA was extracted by the DNeasy Tissue Kit (Qiagen, Hilden, Germany), following the standard procedure. The complete mitochondrion sequence was amplified by PCR with distinct primers and assembled by DNAstar v7.1. MEGA 7.0 software was used for calculating the base composition and constructing a maximum likelihood (ML) tree (Kumar et al. 2016).

The mitogenome of *S. integrilabiatus* was a circular molecule of 16,588 bp in length (GenBank Accession No. MG280782). It consists of 13 protein-coding genes (PCGs), 2 ribosomal RNAs (rRNAs), 22 transfer RNAs (tRNAs), and two non-coding regions: origin of light-strand replication (OL) and control region. The base composition of *S. integrilabiatus* is T 25.6%, C 26.9%, A 30.1%, and G 17.4%. The A + T (55.7%) content is higher than G + C (44.3%) content, which is similar to other fishes (Zhang et al. 2017). Except for eight tRNA (Gln, Ala, Asn, Cys, Tyr, Ser, Glu, and Pro) genes and one PCG (ND6), most of the genes were encoded on the heavy strand (H-strand) which again shows a typical gene arrangement of vertebrate mitogenomes (Anderson et al. 1981).

The phylogenetic tree (Figure 1) was constructed by the maximum-likelihood methods using complete mitochondrial genomes of 16 Schizothoracinae species. The tree supports clear phylogenetic relationships at the genus level. This work provides additional molecular information for studying *S. integrilabiatus* conservation genetics and evolutionary relationships.

**Figure 1.**
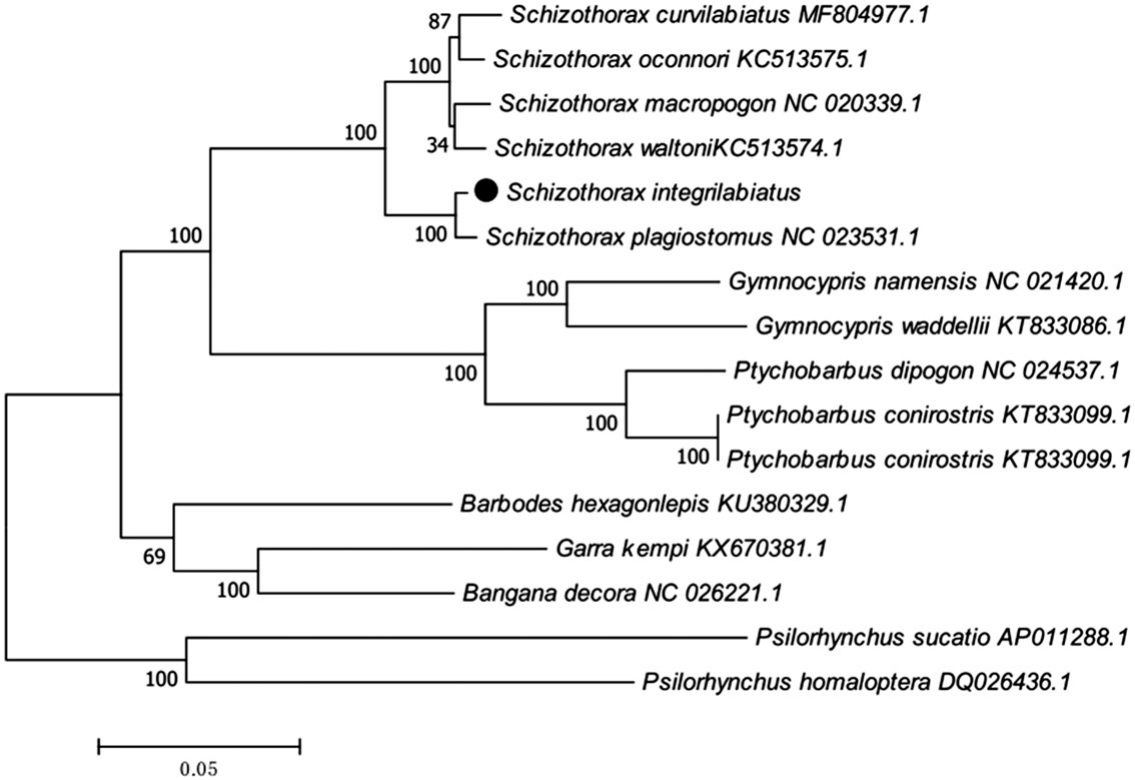
Phylogenetic tree based on the complete mitochondrial genome sequences was constructed by maximum likelihood (ML) analysis with Kimura 2-parameter method with 500 bootstrap replicates.
